# Supplementary material for: ZHX2 promotes HIF1α oncogenic signaling in triple-negative breast cancer
Source: eLife. 2021 Nov 15;10:e70412. doi: 10.7554/eLife.70412 (PMC8673836; doi:10.7554/eLife.70412)

Figure 1—source data. Uncropped western blot images for Figure 1

Figure 1E

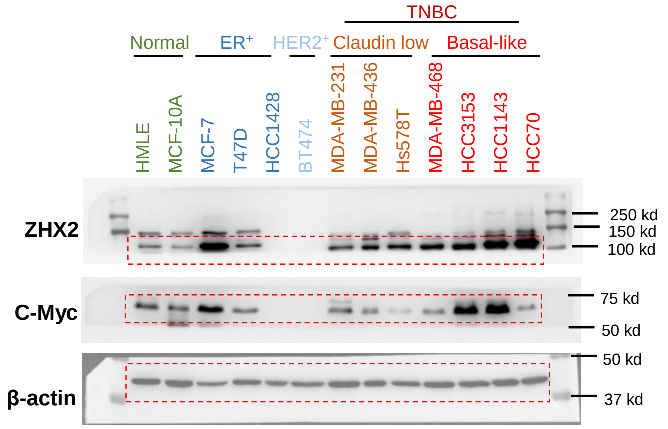

Figure 1F

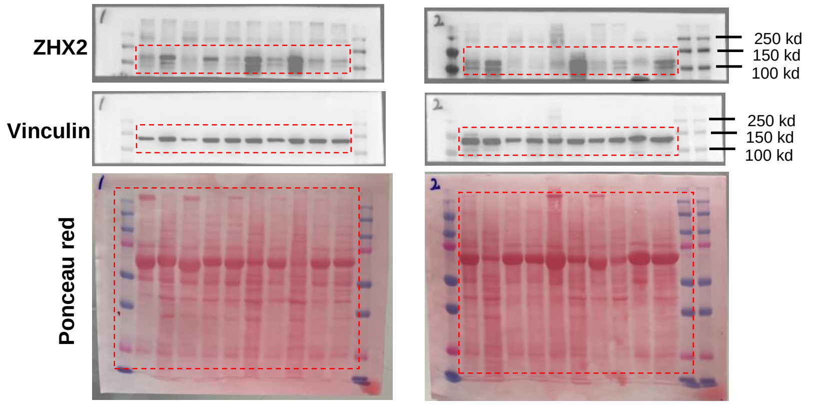

Figure 1I

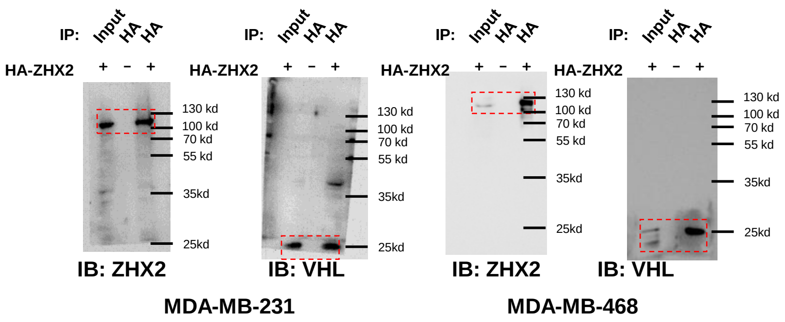

Figure 1J

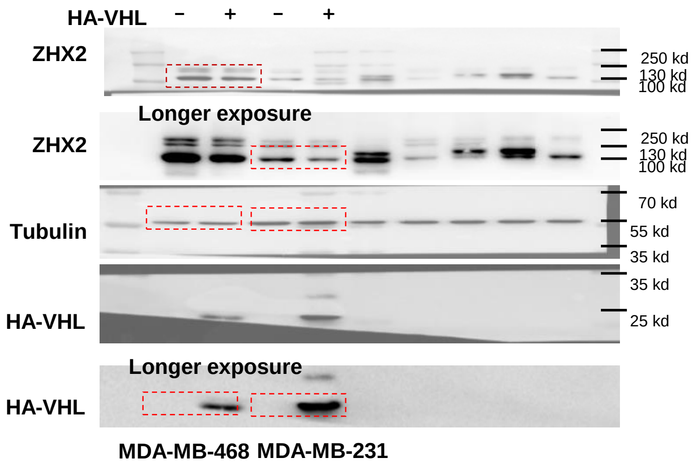

Figure 1K

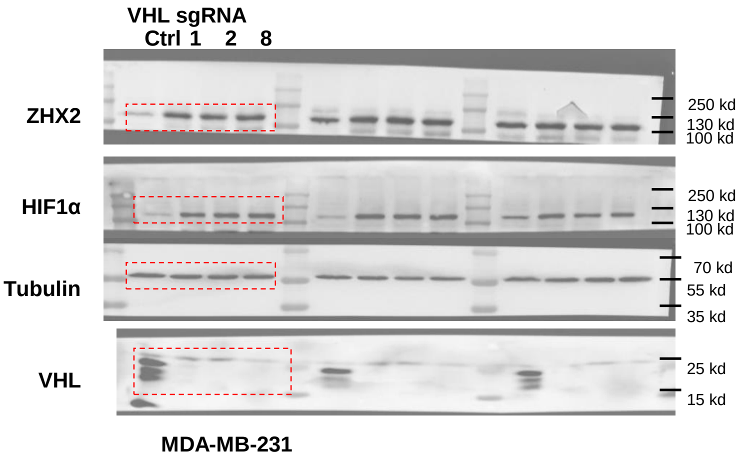

Figure1L

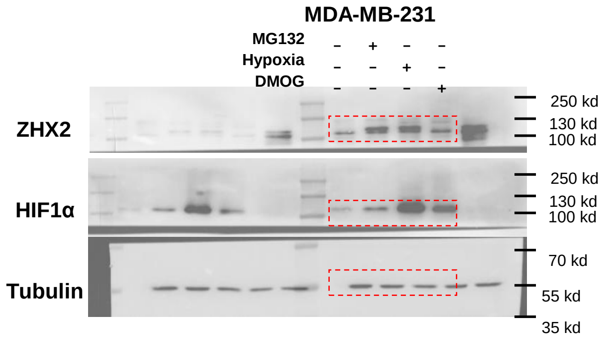

Supplement: Figure 1—source data 1. [file elife-70412-fig1-data1.pdf]
