# Supplementary figures and images for: ZHX2 promotes HIF1α oncogenic signaling in triple-negative breast cancer
Source: eLife. 2021 Nov 15;10:e70412. doi: 10.7554/eLife.70412 (PMC8673836; doi:10.7554/eLife.70412)

**Figure 2—source data.** Uncropped western blot images for Figure 2

**Figure 2A**

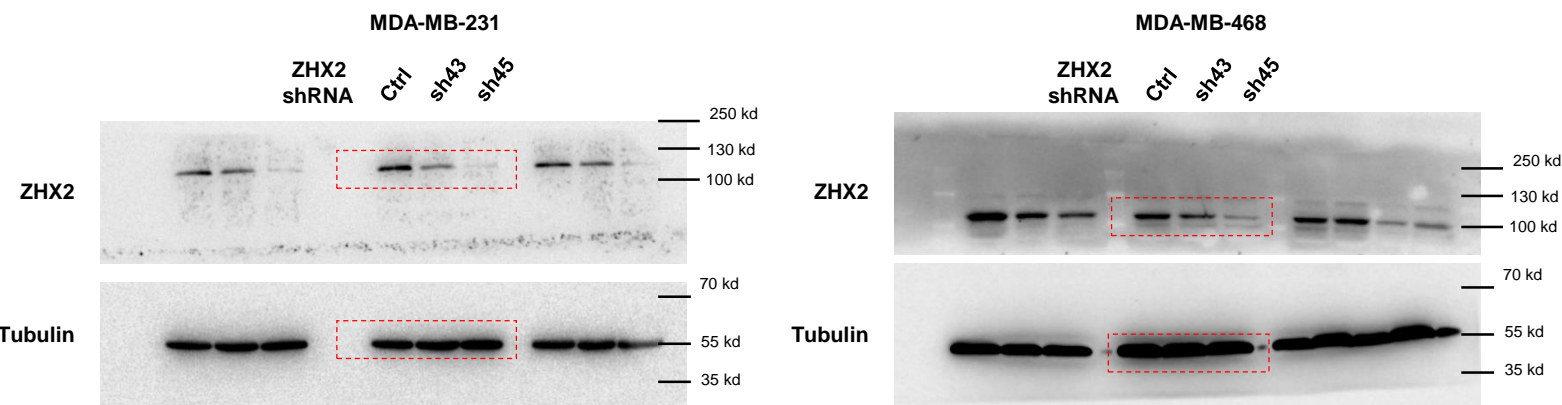

**Figure 2I**

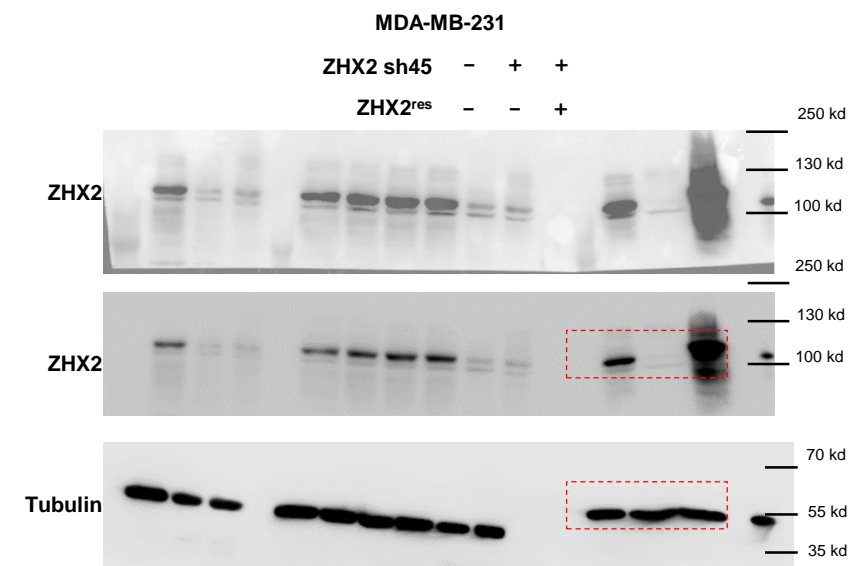

Supplement: Figure 2—source data 1. [file elife-70412-fig2-data1.pdf]

**Figure 3—source data.** Uncropped western blot images for Figure 3

**Figure 3A**

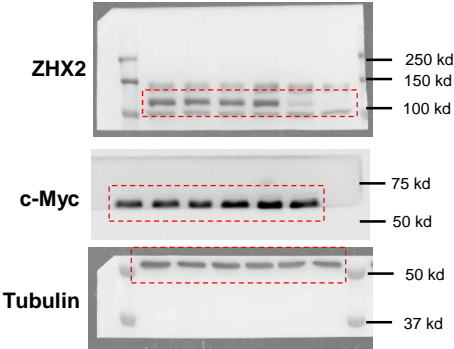

**Figure 3E**

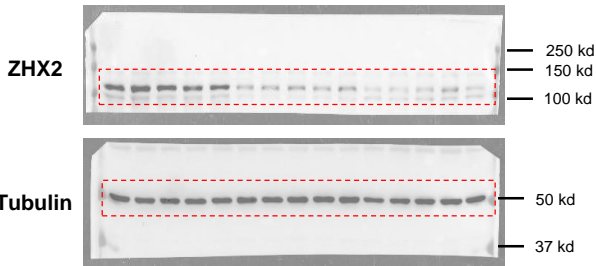

**Figure 3H**

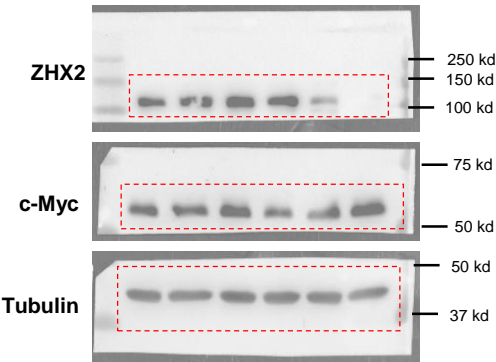

Supplement: Figure 3—source data 1. [file elife-70412-fig3-data1.pdf]

# Figure 4—source data. Uncropped western blot images for Figure 4

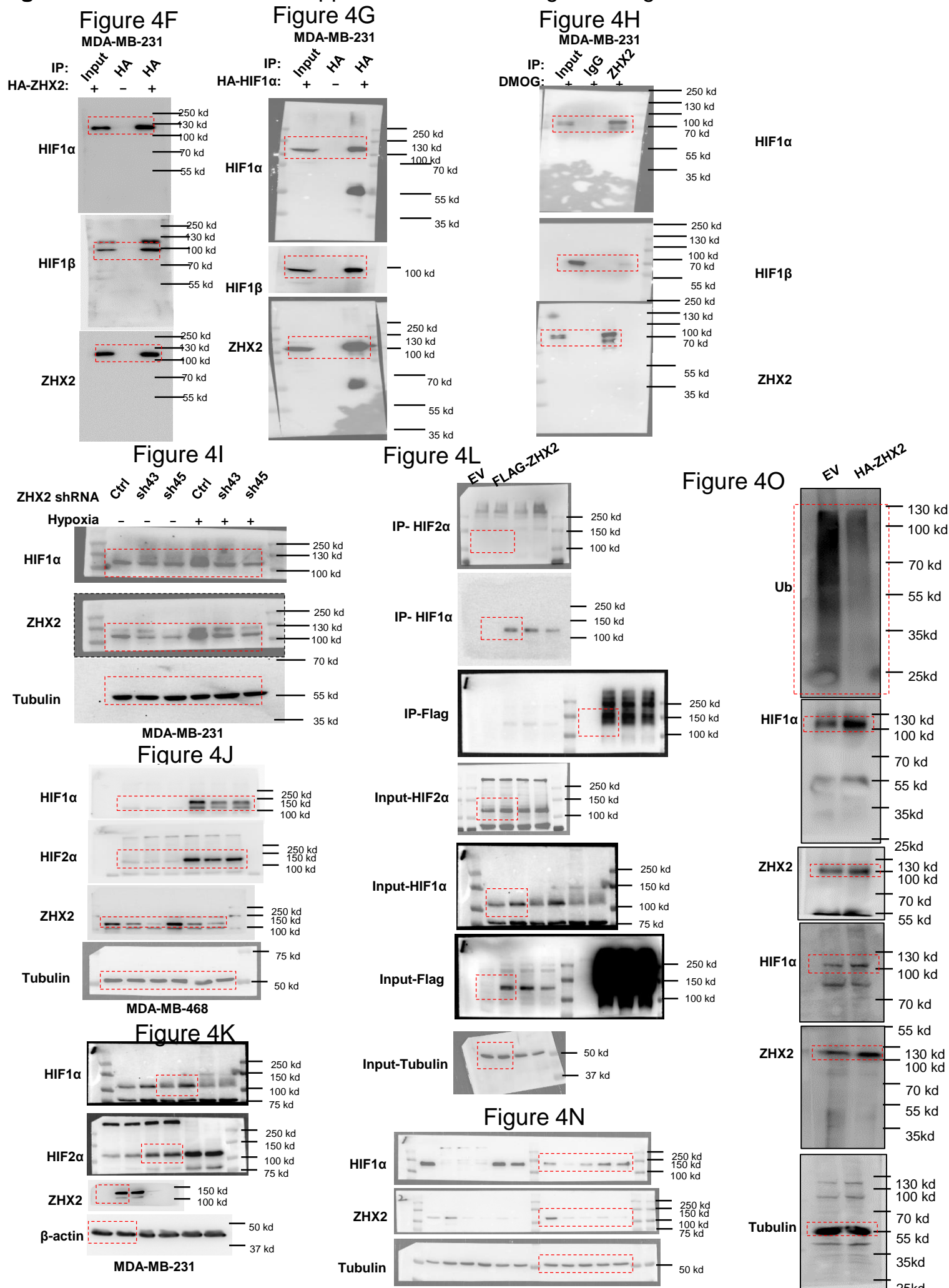

Supplement: Figure 4—source data 1. [file elife-70412-fig4-data1.pdf]

**Figure 5—source data.** Uncropped western blot images for Figure 5

Figure 5E

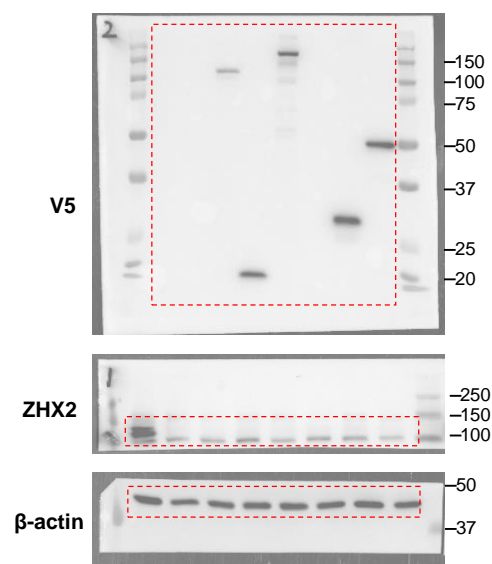

Supplement: Figure 5—source data 1. [file elife-70412-fig5-data1.pdf]

**Figure 6—source data.** Uncropped western blot images for Figure 6

Figure 6A

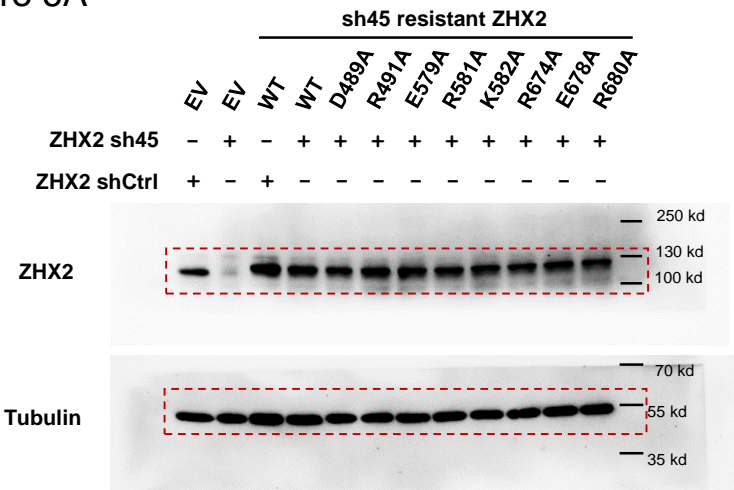

MDA-MB-231

Figure 6G

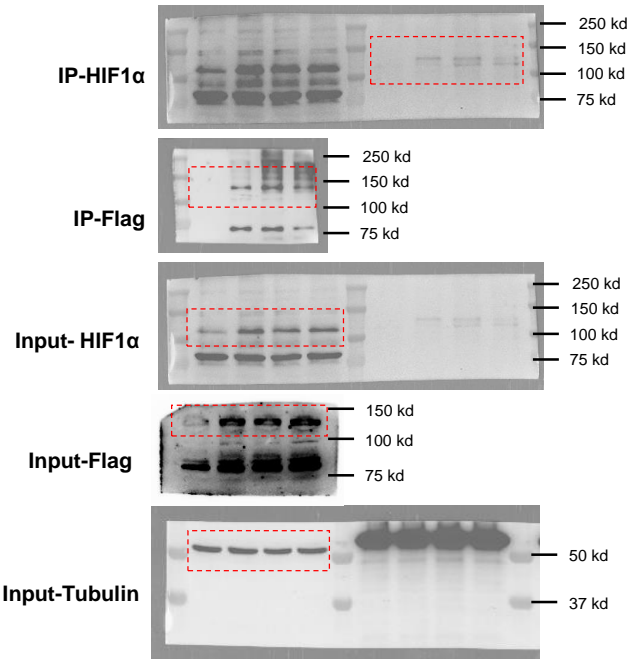

Figure 6H

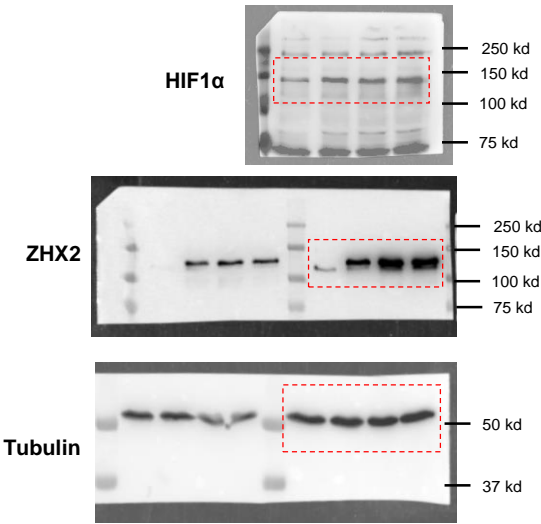

Supplement: Figure 6—source data 1. [file elife-70412-fig6-data1.pdf]
