# Supplementary material for: ZHX2 promotes HIF1α oncogenic signaling in triple-negative breast cancer
Source: eLife. 2021 Nov 15;10:e70412. doi: 10.7554/eLife.70412 (PMC8673836; doi:10.7554/eLife.70412)

Figure 2-figure supplement 1—source data. Uncropped western blot images for Figure 2-figure supplement 1.

figure supplement 1A

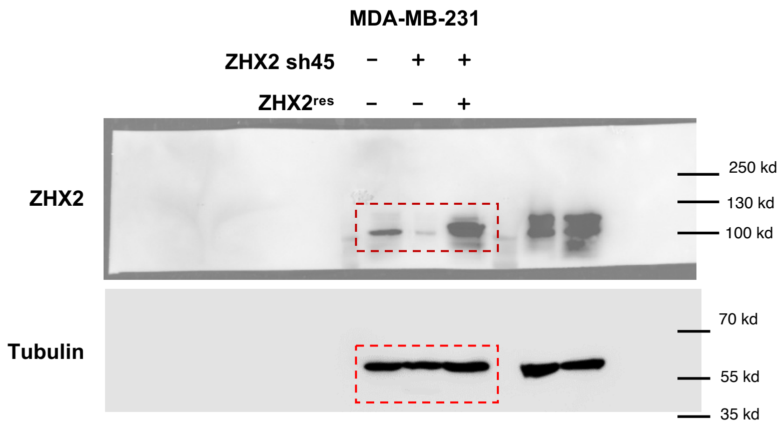

Supplement: Figure 2—figure supplement 1—source data 1. [file elife-70412-fig2-figsupp1-data1.pdf]
