# Supplementary material for: ZHX2 promotes HIF1α oncogenic signaling in triple-negative breast cancer
Source: eLife. 2021 Nov 15;10:e70412. doi: 10.7554/eLife.70412 (PMC8673836; doi:10.7554/eLife.70412)

Figure 4-figure supplement 2—source data. Uncropped western blot images for Figure 4-figure supplement 2.

figure supplement 2G

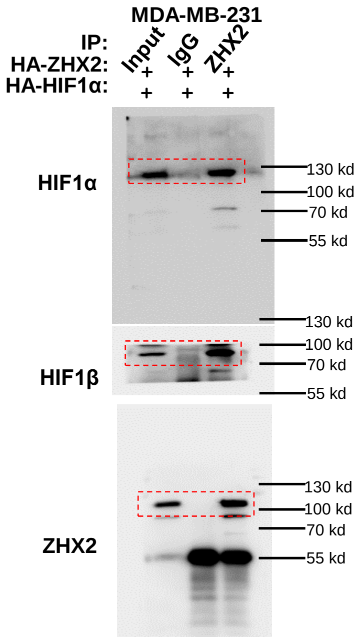

figure supplement 2H

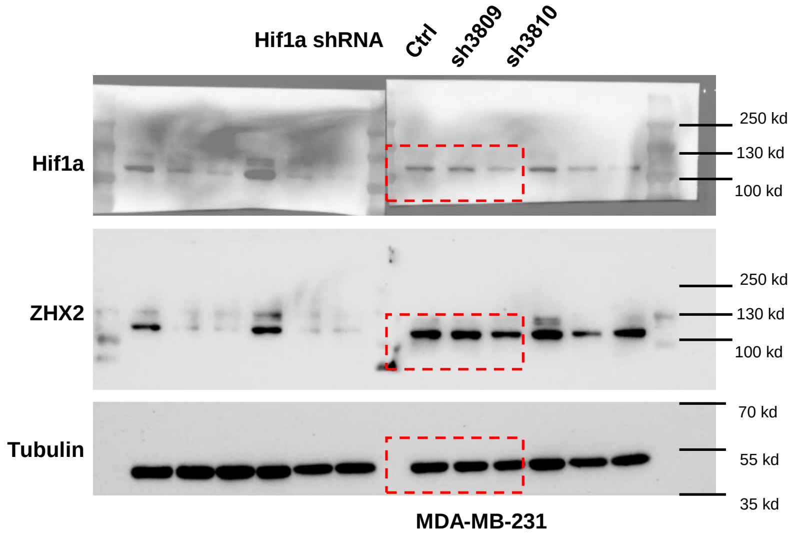

Supplement: Figure 4—figure supplement 2—source data 1. [file elife-70412-fig4-figsupp2-data1.pdf]
