# Supplementary material for: ZHX2 promotes HIF1α oncogenic signaling in triple-negative breast cancer
Source: eLife. 2021 Nov 15;10:e70412. doi: 10.7554/eLife.70412 (PMC8673836; doi:10.7554/eLife.70412)
Supplement: Supplementary file 1. — (a) Amplification status of ZHX2 in different breast cancer subtype. (b) The clinical information of the TNBC patient samples. (c) Top DNA-contacting residues in HD2/3/4 along with their evolutionary conservation. (d) MM/PBSA Calculations of the Free Energy (ΔH-TΔS) for Homeobox 2-4 dsDNA complexes. (e) Top C-terminal helix residues in the Homeobox 2-4 contributing the most DNA binding enthalpy. (f) Real-time PCR primers used in this study. [file elife-70412-supp1.docx]

**Supplemental Files**

**Supplementary File 1a.** **Amplification status of ZHX2 in different breast cancer subtype.**

| Dataset | Breast cancer patients | | | | TNBC patients | | | | ER positive | | | | HER2 positive | | | |
| --- | --- | --- | --- | --- | --- | --- | --- | --- | --- | --- | --- | --- | --- | --- | --- | --- |
|  | Patient | Sample | ZHX2 Amp | Percentage | Patient | Sample | ZHX2 Amp | Percentage | Patient | Sample | ZHX2 Amp | Percentage | Patient | Sample | ZHX2 Amp | Percentage |
| Breast Cancer (METABRIC, Nature 2012 & Nat Commun 2016) | 2509 | 2509 | 512 | 23.6 | 320 | 320 | 104 | 32.5 | 1825 | 1825 | 341 | 21.1 | 247 | 247 | 81 | 32.8 |
| The Metastatic Breast Cancer Project (Provisional, February 2020) | 180 | 237 | 44 | 18.6 | 5 | 6 | 2 | 33.3 | 94 | 126 | 18 | 14.3 | 37 | 44 | 10 | 22.7 |
| Breast Invasive Carcinoma (TCGA, Cell 2015) | 817 | 818 | 148 | 18.1 | 83 | 83 | 25 | 30.1 | 601 | 601 | 90 | 15.0 | 121 | 121 | 31 | 25.6 |
| Breast Invasive Carcinoma (TCGA, Firehose Legacy) | 1101 | 1108 | 197 | 18.2 | 116 | 117 | 36 | 32.1 | 808 | 814 | 120 | 15.1 | 164 | 164 | 39 | 24.1 |
| Breast Invasive Carcinoma (TCGA, Nature 2012) | 825 | 825 | 92 | 11.8 | 123 | 123 | 23 | 19.3 | 601 | 601 | 58 | 10.3 | 114 | 114 | 20 | 18.2 |

**Supplementary File 1b.** **The clinical information of the TNBC patient samples.**

| Patient | Gender | Tissue description | Anatomic site | Case diagnosis | HER2 IHC | PR | ER |
| --- | --- | --- | --- | --- | --- | --- | --- |
| #1 | Female | Tumor-primary | Breast | Infiltrating duct carcinoma, NOS | Negative | Negative (<1% or stated as negative) | Negative (<1% or stated as negative) |
| #2 | Female | Tumor-primary | Breast | Infiltrating duct carcinoma, NOS | Negative | Negative (<1% or stated as negative) | Negative (<1% or stated as negative) |
| #3 | Female | Tumor-primary | Breast | Infiltrating duct carcinoma, NOS | Negative | Negative (<1% or stated as negative) | Negative (<1% or stated as negative) |
| #4 | Female | Tumor-primary | Breast | Infiltrating duct carcinoma, NOS | Negative | Negative (<1% or stated as negative) | Negative (<1% or stated as negative) |
| #5 | Female | Tumor-primary | Breast | Infiltrating duct carcinoma, NOS | Negative | Negative (<1% or stated as negative) | Negative (<1% or stated as negative) |
| #6 | Female | Tumor-primary | Breast | Infiltrating duct carcinoma, NOS | Negative | Negative (<1% or stated as negative) | Negative (<1% or stated as negative) |
| #7 | Female | Tumor-primary | Breast | Infiltrating duct carcinoma, NOS | Negative | Negative (<1% or stated as negative) | Negative (<1% or stated as negative) |
| #8 | Female | Tumor-primary | Breast | Infiltrating duct carcinoma, NOS | Negative | Negative (<1% or stated as negative) | Negative (<1% or stated as negative) |
| #9 | Female | Tumor-primary | Breast | Infiltrating duct carcinoma, NOS | Negative | Negative (<1% or stated as negative) | Negative (<1% or stated as negative) |
| #10 | Female | Tumor-primary | Breast | Infiltrating duct carcinoma, NOS | Negative | Negative (<1% or stated as negative) | Negative (<1% or stated as negative) |
| #11 | Female | Tumor-primary | Breast | Infiltrating duct carcinoma | Negative | Negative (<1% or stated as negative) | Negative (<1% or stated as negative) |
| #12 | Female | Tumor-primary | Breast | infiltrating carcinoma | Negative | Negative (<1% or stated as negative) | Negative (<1% or stated as negative) |
| #13 | Female | Tumor-primary | Breast | infiltrating carcinoma | Negative | Negative (<1% or stated as negative) | Negative (<1% or stated as negative) |
| #14 | Female | Tumor-primary | Breast | Infiltrating duct carcinoma | Negative | Negative (<1% or stated as negative) | Negative (<1% or stated as negative) |
| #15 | Female | Tumor-primary | Breast | Infiltrating duct carcinoma | Negative | Negative (<1% or stated as negative) | Negative (<1% or stated as negative) |
| #16 | Female | Tumor-primary | Breast | infiltrating carcinoma | Negative | Negative (<1% or stated as negative) | Negative (<1% or stated as negative) |
| #17 | Female | Tumor-primary | Breast | Infiltrating duct carcinoma | Negative | Negative (<1% or stated as negative) | Negative (<1% or stated as negative) |
| #18 | Female | Tumor-primary | Breast | Infiltrating duct carcinoma | Negative | Negative (<1% or stated as negative) | Negative (<1% or stated as negative) |
| #19 | Female | Tumor-primary | Breast | Infiltrating duct carcinoma | Negative | Negative (<1% or stated as negative) | Negative (<1% or stated as negative) |
| #20 | Female | Tumor-primary | Breast | Infiltrating duct carcinoma | Negative | Negative (<1% or stated as negative) | Negative (<1% or stated as negative) |

**Supplementary File 1c.**  **Top DNA-contacting residues in HD2/3/4 along with their evolutionary conservation.**

| **Homeobox 2** | | | **Homeobox 3** | | | **Homeobox 4** | | |
| --- | --- | --- | --- | --- | --- | --- | --- | --- |
| **Residue** | **Mean Total Contacts** | **Conserv-ation Score*** | **Residue** | **Mean Total Contacts** | **Conserv-ation Score*** | **Residue** | **Mean Total Contacts** | **Conserv-ation Score*** |
| LYS 485 | 52.50 | 5 | **GLU 579** | 31.05 | 8 | **ARG 674** | 129.20 | 6 |
| **ARG 491** | 36.33 | 9 | **ARG 581** | 24.80 | 6 | **GLU 678** | 58.73 | 8 |
| PHE 463 | 32.92 | 2 | SER 575 | 23.20 | 5 | GLU 671 | 45.80 | 7 |
| ARG 493 | 25.08 | 5 | **LYS 582** | 23.00 | 7 | **ARG 680** | 28.33 | 9 |
| **ASP 489** | 22.42 | 8 | PHE 553 | 13.00 | 1 | LYS 635 | 27.73 | 9 |
| GLU 482 | 14.25 | 6 | GLU 572 | 9.55 | 8 | LYS 684 | 25.13 | 7 |
| LYS 484 | 11.42 | 7 | ARG 570 | 9.25 | 7 | LYS 677 | 23.47 | 7 |
| TYR 492 | 9.58 | 5 | TRP 576 | 7.55 | 9 | TRP 652 | 15.53 | 4 |
| ARG 480 | 8.67 | 8 | ASP 585 | 4.70 | 7 | TRP 675 | 10.13 | 9 |
| TRP 486 | 7.42 | 9 | SER 578 | 4.70 | 5 | CYS 681 | 6.33 | 7 |

*obtained using ConSurf server (9 – most conserved, 1 – most diverse); bold-faced residues were chosen

for the mutagenesis study on TNBC suppression. DNA-protein contact is defined by heavy atom contact within 4Å, while the same DNA atoms contacted by different protein atoms are considered as separate contacts.

**Supplementary File 1d. MM/PBSA Calculations of the Free Energy (ΔH-TΔS) for Homeobox 2-4 dsDNA complexes.**

|  | **ΔH (kcal/mol)** | **(TΔS) (kcal/mol)** | **ΔG (kcal/mol)** |
| --- | --- | --- | --- |
| **HD2+DNA** | -60.25 ± 0.50 | -48.05 ± 0.97 | **-12.20 ± 1.09** |
| **HD3+DNA** | -56.93 ± 0.46 | -45.53 ± 2.39 | **-11.40 ± 2.43** |
| **HD4+DNA** | -39.00 ± 0.50 | -31.23 ± 2.34 | **-7.77 ± 2.39** |

**Supplementary File 1e. Top C-terminal helix residues in the Homeobox 2-4 contributing the most DNA binding enthalpy.**

| **Homeobox 2** | | **Homeobox 3** | | **Homeobox 4** | |
| --- | --- | --- | --- | --- | --- |
| **Residue number**** | **ΔH_total_ (kcal/mol)*** | **Residue number**** | **ΔH_total_ (kcal/mol)*** | **Residue number**** | **ΔH_total_ (kcal/mol)*** |
| ARG 491 | -8.75 ± 0.17 | ARG 581 | -12.51 ± 0.15 | ARG 674 | -6.81 ± 0.12 |
| ARG 480 | -7.98 ± 0.08 | ARG 571 | -7.54 ± 0.09 | LYS 684 | -3.46 ± 0.08 |
| ARG 493 | -7.41 ± 0.09 | LYS 582 | -6.98 ± 0.10 | LYS 677 | -3.18 ± 0.07 |
| LYS 484 | -6.49 ± 0.08 | ARG 580 | -4.43 ± 0.02 | ARG 680 | -2.41 ± 0.01 |
| LYS 485 | -5.00 ± 0.11 | ARG 570 | -2.66 ± 0.02 | ARG 669 | -1.78 ± 0.01 |
| TYR 492 | -2.88 ± 0.03 | ARG 584 | -2.24 ± 0.01 | TRP 675 | -1.45 ± 0.05 |
| ARG 496 | -2.12 ± 0.11 | SER 578 | -0.71 ± 0.06 | LEU 682 | -0.79 ± 0.02 |
| GLN 495 | -1.86 ± 0.07 | PHE 577 | -0.35 ± 0.01 | CYS 681 | -0.36 ± 0.02 |
| TRP 486 | -0.96 ± 0.06 | LEU 583 | -0.22 ± 0.00 | THR 670 | -0.36 ± 0.04 |
| HID 490 | -0.64 ± 0.01 |  |  | ASN 679 | -0.24 ± 0.02 |
| SER 481 | -0.49 ± 0.03 |  |  |  |  |
| PHE 487 | -0.28 ± 0.01 |  |  |  |  |
| CYS 494 | -0.21 ± 0.00 |  |  |  |  |

* ΔH_total_: enthalpic binding energy derived from MM/PBSA, **this numbering is based on the Uniprot database where the sequence was obtained, highlighted in blue represents the mutated amino acid in the experimental results

**Supplementary File 1f. Real-time PCR primers used in this study.**

| **Genes** | **Forward Primer** | **Reverse Primers** |
| --- | --- | --- |
| *β-Actin* | AGAAAATCTGGCACCACACC | GGGGTGTTGAAGGTCTCAAA |
| *ZHX2* | GATCAGATAGCTGGAGTCAGGC | CACAGCAGTTCTAACAGACTTCC |
| *TMEM45A* | TCCTCTCCTTCTCGCCACTT | TGTGTTGGATGGGATCTGGC |
| *PNRC1* | TGTTCCGCGATCTTCTCAGG | GCTAGGAAGCTTGTCGCTCA |
| *CCNG2* | AGTGATTCCAGAGTGAGCCTT | AAGGCACAGATGCCAAACCTA |
| *ALDOC* | CTGCAGCCTCATCTGTTTGC | CATGGTGACAGCTCCCTGTG |
| *AKAP12* | CGAGCGCGTCTCCTTCATT | GGGCAAGAGCCAAAAGACG |
| *ADM* | ATGAAGCTGGTTCCCGTAGC | TCCACGACTTAGAGCCCACT |
| *NDRG1* | CTGCACCTGTTCATCAATGC | AGAGAAGTGACGCTGGAACC |
| *BNIP3* | CGCAGACACCACAAGATACCA AC | GCCAGCAAATGAGAGAGCAGC |
| *PTGES3L* | GTGTTGAGGACAGCACCGAT | ACACTGGCTTGGAGTTCACTT |
| *KDM3A* | GTGCTCACGCTCGGAGAAA | GTGGGAAACAGCTCGAATGGT |
| *WSB1* | GGTGTCAGCTTCAAGAGACAAA | AGTCAGGAGAGAATGCACAGC |
| *AP2B1* | CTCTTTCCAGACGTAGTGAACTG | GGAGCGGCTCACAGAGATATT |
| *OXSR1* | AGGGACGATTACGAGCTGC | TCCGTTTGATTGCCACTTTCTC |
| *RUNDC1* | AAGAGGGCAGTTATGACTCGC | GCTGGGTTGACGATCTGAGC |
| *COX20* | TAGGATCTGTTGTGGCTGGC | CCAGCATCCCAAAGTCACCA |
| *HIF1α* | TATGAGCCAGAAGAACTTTTAGGC | CACCTCTTTTGGCAAGCATCCTG |
